# Porous Carbon Interlayer Derived from Traditional Korean Paper for Li–S Batteries

**DOI:** 10.3390/nano14040385

**Published:** 2024-02-19

**Authors:** Yunju Choi, Hyungil Jang, Jong-Pil Kim, Jaeyeong Lee, Euh Duck Jeong, Jong-Seong Bae, Heon-Cheol Shin

**Affiliations:** 1 Busan Center, Korea Basic Science Institute (KBSI), Busan 46742, Republic of Korea; yjchoi0512@kbsi.re.kr (Y.C.); edjeong@kbsi.re.kr (E.D.J.); 2 Department of Materials Science and Engineering, Pusan National University, Busan 46241, Republic of Korea

**Keywords:** Korean paper, hanji, lithium–sulfur battery, sulfur cathode, electrochemical performance

## Abstract

A carbonized interlayer effectively helps to improve the electrochemical performance of lithium–sulfur (Li–S) batteries. In this study, a simple and inexpensive carbon intermediate layer was fabricated using a traditional Korean paper called “hanji”. This carbon interlayer has a fibrous porous structure, with a specific surface area of 91.82 m^2^ g^−1^ and a BJH adsorption average pore diameter of 26.63 nm. The prepared carbon interlayer was utilized as an intermediary layer in Li–S batteries to decrease the charge-transfer resistance and capture dissolved lithium polysulfides. The porous fiber-shaped carbon interlayer suppressed the migration of polysulfides produced during the electrochemical process. The carbon interlayer facilitates the adsorption of soluble lithium polysulfides, allowing for their re-utilization in subsequent cycles. Additionally, the carbon interlayer significantly reduces the polarization of the cell. This simple strategy results in a significant improvement in cycle performance. Consequently, the discharge capacity at 0.5 C after 150 cycles was confirmed to have improved by more than twofold, reaching 230 mAh g^−1^ for cells without the interlayer and 583 mAh g^−1^ for cells with the interlayer. This study demonstrates a simple method for improving the capacity of Li–S batteries by integrating a functional carbon interlayer.

## 1. Introduction

Lithium–sulfur (Li–S) batteries are gaining prominence as a leading contender for next-generation energy storage systems. Particularly, compared with lithium-ion batteries (LIBs), sulfur, the cathode material in Li–S batteries, has the advantage of being one of the most abundant elements on Earth, making it highly cost-effective. Additionally, owing to their environmental friendliness and a high theoretical energy density of 1600 Wh kg^−1^, Li–S batteries are considered potential successors in next-generation battery systems [1,2]. However, several challenges hinder the practical application of Li–S batteries. First, sulfur, which serves as the cathode material in Li–S batteries, and the resulting polysulfides formed during reduction, are insulating materials, presenting a challenge for improving conductivity. Secondly, issues related to the expansion and contraction of the battery volume during charging and discharging are challenging. The stepwise reduction in polysulfides formed during the discharge results in volume expansion. This expansion and contraction result in a loss of contact between electrically insulating sulfur and polysulfides, accelerating battery degradation. Thirdly, internal reactions occur in the battery because of the dissolution of polysulfides. Polysulfides persist in the cathode, blocking the passage for lithium ions, increasing internal resistance, and causing a continuous accumulation that contaminates the separator [3,4,5,6,7,8,9]. To address these issues, various studies have been conducted in recent years. To maintain high energy density and stable cycling performance, retaining sulfur content in the cathode material and creating various shapes through nanoscale and nanostructure chemical synthesis is essential. This approach leads to the development of diverse characteristics based on their forms. Porous carbon and carbon nanotubes, known for their excellent electrical conductivity, extensive surface area, and resilience to external stress, are fundamental materials currently being actively researched for the synthesis of various composite materials with sulfur [10]. Additionally, a structure is required to confine the polysulfides generated during the oxidation–reduction reaction of solid sulfur in a ring structure within the cathode. This is necessary to prevent their movement to the anode, thereby enhancing the performance of the Li–S system. Separators used in LiBs must have characteristics such as insolubility in liquid electrolytes, high ion conductivity, and mechanical strength, while also being electrically insulating. In addition to these general characteristics of separators in LiBs, chemical stability with polysulfides and lithium metal (anode) is crucial for Li–S batteries. However, the high reactivity of polysulfides and lithium metal limits the selection of materials for these substances. Several studies have used different interlayers to enhance the performance of Li–S systems for commercializing Li–S batteries. Metal-oxide coatings can be used to fabricate interlayers on commercial separators, such as V_2_O_5_ [11], Al_2_O_3_, and TiO_2_ [12]. Carbon materials have also been used, such as graphene [13,14], conductive carbon black, multiwalled carbon nanotubes [15,16], microporous carbon paper [17], carbon paper interlayers [18,19], carbon nanoforms [20], vanadium nitride quantum dots, and hollow nitrogen–carbon nanocages [21].

Polymeric binders are suitable for forming carbon interlayers; further, carbon nanotubes and fibers are relatively expensive. The synthesis of carbon materials from low-cost and environmentally friendly biomass has recently received considerable attention [22,23]. These materials have been used as the interlayer in Li–S battery systems [24], and one study showed that carbon filter paper can be fabricated through pyrolysis and used as an interlayer in Li–S batteries [25].

Hanji, a type of paper, is a traditional Korean paper made from the bark of the paper mulberry tree (*Broussonetia kazinoki Sieb*.), collected and processed to create traditional Korean paper. The bark of the mulberry tree is collected, processed, and used to prepare hanji. The paper manufacturing process involves many steps, including steaming, debarking, creating white fabric, drying, washing, bleaching, tapping, dissociation, papermaking, and elimination of nonfibrous materials using an alkaline solution. Hanji is a strong and porous paper, and it is used in wallpaper, window paper, napkins, nicotine-elimination filters, and high-quality wrapping paper, among other applications [26]. Hanji is a widely used mass-produced low-cost biomass. Additionally, because the paper sheet is monolithic, it may be converted into carbon-fiber paper using a simple carbonization method. According to previously reported research using a similar approach, bamboo paper [24] and Chinese rice paper (also known as Xuan paper) [27] have been presented as interlayers for Li-S batteries. However, this study differs from previous publications by excluding the carbon coating process and conducting only heat treatment, which simplifies the process. Hence, in this study, we used hanji to enhance the electrochemical performance of Li–S batteries. A carbon interlayer paper was prepared in a simple and low-cost process as a porous fiber material. By utilizing active materials more effectively, the cell design with hanji as an interlayer can reduce the resistance of the sulfur cathode.

## 2. Materials and Methods

### 2.1. Preparation of Carbon Interlayer

The hanji was purchased from Jeonju City (Jeollabukdo Province, Republic of Korea) and washed with deionized water to remove impurities and dust adhering to its surface. The samples were then dried. The hanji–carbon interlayer (HC−In) was carbonized at 800 °C for 1 h at a heating rate of 5 °C min^−1^ in a tube furnace. Carbonization was conducted under a N_2_ gas flow rate of 200 mL min^−1^. An interlayer was used to prevent the diffusion of Li_x_S toward the anode. The interlayer was of the same size (1.9 cm) as the separator, with a weight of approximately 6.5 mg. The thickness of the HC−In was approximately 67 μm. Through the carbonization process of the HC−In, the carbon yield was observed to reach approximately 74% when the temperature was raised up to 800 °C. Approximately 26% of the material underwent carbonization and was consequently lost or eliminated in the process.

### 2.2. Preparation of Flake C-Coated Separator

A flake carbon-coated separator (FCCS) was prepared as follows. Into a slurry containing 0.2 g of flake-type graphite, polyvinylidene fluoride (PVDF) was dissolved, with a flake carbon/PVDF weight ratio of 90 wt%:10 wt%. This was coated onto a polypropylene membrane separator (Celgard 2400, Charlotte, North Carolina, USA, 25 μm thick) using *N*–methyl–2–pyrrolidone (NMP). After drying this at room temperature for 1 h, further drying was conducted in an oven at 50 °C for more than 5 h. The dried separator film was then roll-pressed to prepare the FCCS and cut to a diameter of 1.9 cm [28].

### 2.3. Material Characterization

The surface areas and pore sizes of the porous carbon composites were estimated using the Brunauer–Emmett–Teller (BET) and Barrett–Joyner–Halenda (BJH) methods, respectively. X-ray diffraction (XRD; PANalytical Malvern, Worcesterchire, UK, 40 kV, 15 mA) patterns were recorded using Cu Kα radiation with λ = 1.5406 Å. The measurement range was set from 10° to 80° at a rate of 3°/min, and the crystalline phases of the prepared samples were confirmed. The morphologies and structure of the samples were observed using scanning electron microscopy (SEM; SU-70, Hitachi, Tokyo, Japan, at accelerating voltages of 30 and 200 kV) and field-emission transmission electron microscopy (FE-TEM; JEM-2100F, JEOL, Akishima, Japan, operated at 200 kV) with element-mapping system functionality.

### 2.4. Electrochemical Measurements 

Electrochemical properties were measured using CR2032 coin-type cells assembled in an Ar-filled glove box with Li foil as the counter electrode. For the working electrode, a mixture of the active material, carbon black (Super P), and polyvinylidene difluoride (Solef^®^ 5130, Solvay, Tavaux, France, 4 wt% dissolved in NMP) binder with a mass ratio of 70 wt%:20 wt%:10 wt% (JUNKEI) was coated on an Al foil current collector using a doctor blade. The coated electrode was dried in an oven at 60 °C overnight and then roll-pressed. The resulting electrode had a thickness of ~20 µm with an active material mass loading of ~1.0 mg cm^−2^. Subsequently, the cathode was cut into a pellet with a diameter of 1.4 cm, then dried in an oven at 60 °C for 12 h. The coin cell was assembled in a glove box filled with Ar gas. For the assembly of the CR2032 coin-type half-cell, a carbon composite electrode was used as the working electrode and Li metal served as the counter electrode. As a separator, a 25 µm thick commercial Celgard 2400 polypropylene membrane and HC−In were sandwiched between the working and counter electrodes. Additionally, 1.0 M lithium bis(trifluoromethylsulfonyl)imide and 0.4 M LiNO_3_ in a solvent mixture of dioxolane and dimethoxyethane (1:1 *v*:*v*) were used as the electrolyte. Electrochemical cycling tests were conducted using a galvanostat/potentiostat system (WBCS 3000, WonATech Co., Ltd., Seoul, Republic of Korea) at 35 °C. The tests were performed in a cut-off voltage range of 1.5–2.8 V vs. Li/Li^+^ at 0.5 C and 1 C. Electrochemical impedance spectroscopy (EIS) measurements were performed using potentiostat/galvanostat/EIS systems (ZICE Smart (SM) Software 6, WonATech Co., Ltd., Seoul, Republic of Korea) over a frequency range of 10^−1^ Hz to 10^7^ Hz.

**Figure 1:**
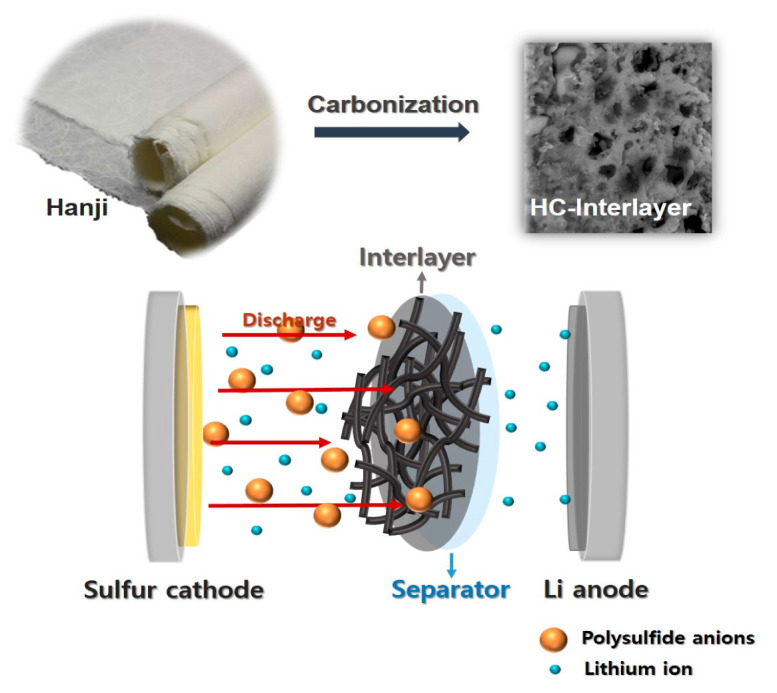
Schematic of Li–S battery with HC interlayer.

The shuttle effect of dissolved lithium polysulfide is a hindrance in Li–S batteries. To address this issue, as illustrated in Figure 1, we introduced an interlayer called HC−In. Serving as an intermediate layer between the separator and the cathode, the HC−In plays a crucial role in preventing polysulfides from reaching the Li anode side. This function is achieved through both physical and chemical interactions, effectively impeding the movement of polysulfides toward the Li anode.

## 3. Results and Discussion

**Figure 2:**
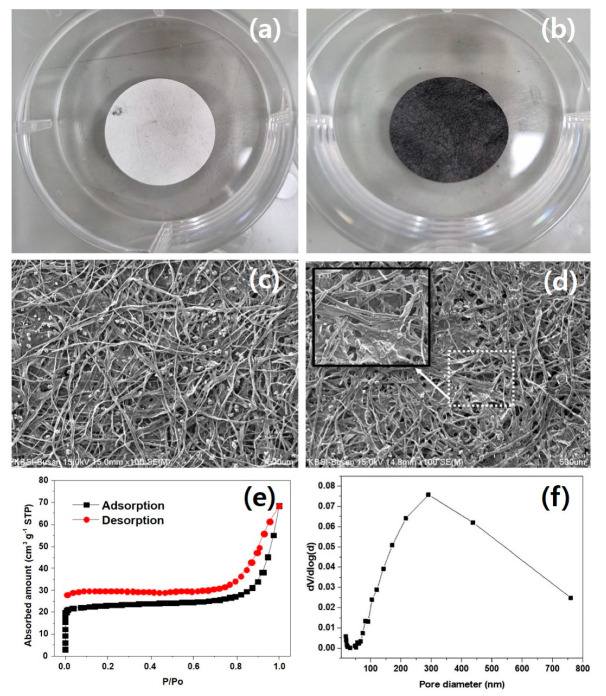
Photographs of (**a**) hanji and (**b**) HC−In; SEM images of (**c**) hanji and (**d**) HC−In, inset shows a porous network-shaped structure (**e**) N2 adsorption/desorption isotherms and (**f**) pore–size distribution of the prepared HC−In.

HC−In is a single handheld carbon paper product produced by the thermal decomposition of Korean paper. Figure 2a shows the state of the Korean paper before thermal treatment, and Figure 2b shows the state of the HC−In after thermal treatment. The SEM images of hanji (Figure 2c) and the HC−In (Figure 2d) show that they are primarily composed of fibers. The inset in Figure 2d shows a porous network-shaped structure that arises due to the loss of elements such as oxygen and hydrogen at high temperatures. The HC−In fiber has a nanosized thickness, enabling contact with the sulfur cathode, thereby reducing the electrical resistance. In Figure 2e,f, the nitrogen adsorption/desorption isotherm of the HC−In shows a BET surface area of 91.82 m^2^ g⁻^1^, and the BJH pore size distribution derived from the N_2_ adsorption branch indicates a diameter of 26.349 nm.

**Figure 3:**
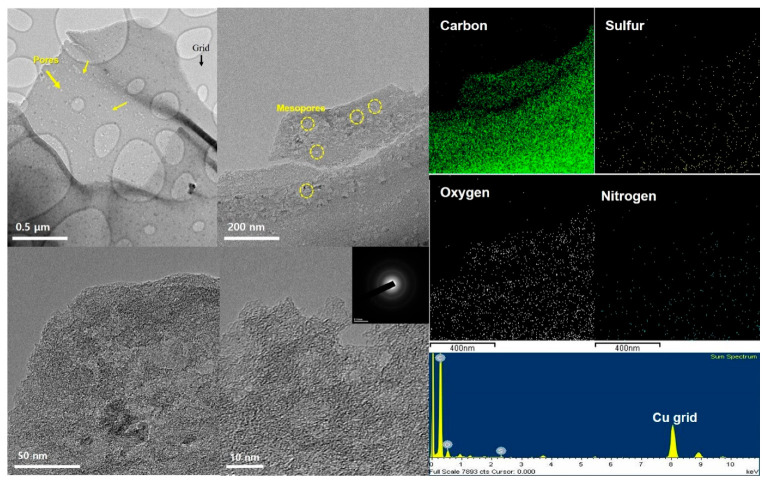
HR-TEM images and elemental mapping of the HC−In.

Figure 3 shows the structural and morphological images of HC−In captured using an electron microscope. The TEM image indicated that it has a smaller secondary pore structure compared with the SEM image. At low magnification, small pores were confirmed to be distributed. Upon closer examination, the results indicated that circular pores with a diameter of approximately 20 nm or less were uniformly distributed throughout the carbon structure. These micrographs appear to align with the BET measurement results. In addition, the components of the HC−In were analyzed using EDS mapping. Therefore, the majority of these components were distributed in carbon and sulfur. This result supports the effective carbonization of hanji.

**Figure 4:**
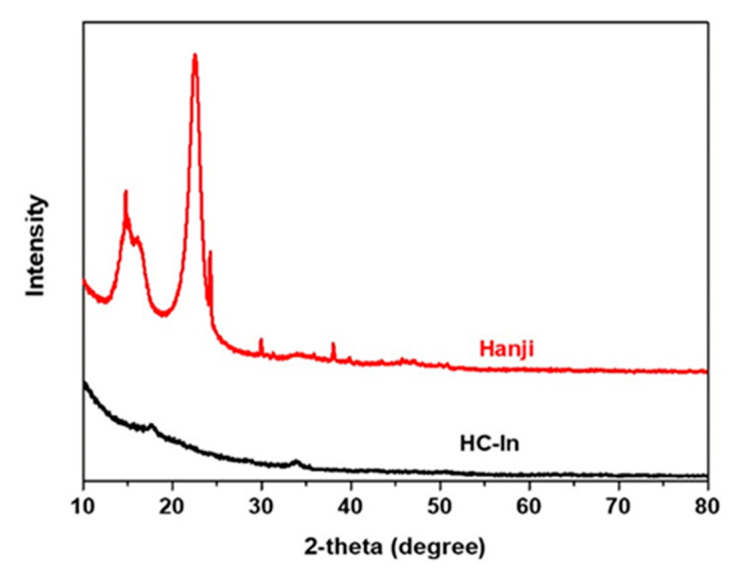
XRD patterns of the porous HC−In and hanji.

Figure 4 shows the XRD patterns of hanji and HC−In before heat treatment. The hanji exhibits a cellulose form prior to heat treatment, while the HC−In is in the form of graphitized carbon after pyrolysis. The XRD pattern of hanji reveals the existence of reflective properties in a hexagonal carbon structure, with distinct diffraction peaks at approximately 24° and 44°. These peaks correspond to the (002) and (100)/(101) planes, respectively, suggesting the presence of a partially amorphous graphitized structure between the carbon layers.

**Figure 5:**
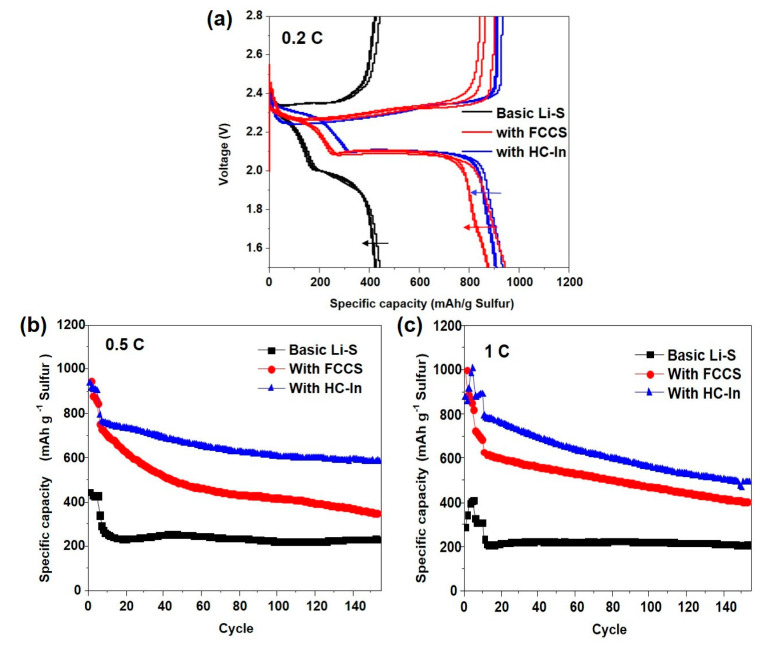
(**a**) Charge–discharge voltage profiles of Li–S cells with FCCS and HC−In in the first, second, and third cycles at a 0.2 C rate; (**b**,**c**) cycling performance at 0.5 C and 1 C.

In this study, we compared the performance of a Li–S battery using the conventional method to assess the battery’s performance with the addition of the FCCS or HC−In. Figure 5a shows the initial charge–discharge voltage with and without the insertion of FCCS and HC−In. When comparing the charge–discharge curves for the first, second, and third cycles at 0.2 C, we observed that the capacities without the HC−In and with the HC−In were 405.08 mAh g^−1^ and 935.26 mAh g^−1^, respectively. This indicates a significant improvement in capacity when the HC−In is used. The HC−In material, composed solely of carbon and coated with carbon at the center of the electrode, contributes to the increase in capacity. During charging and discharging between the Li and S electrode materials, Li_2_S is generated with the FCCS. Consequently, Li_2_S is captured by the FCCS and HC−In materials, resulting in a greater contribution to electron movement compared with that with the existing separator. Additionally, both the FCCS and HC−In not only reduce the internal resistance of the cell but also mechanically block the diffusion of sulfur away from the cathode and into the electrolyte, thereby enhancing sulfur utilization and specific capacity.

Figure 5b,c compare the cycling performances of the Li–S cells using the FCCS and HC−In interlayers, respectively. After each coin cell (0.5 C and 1 C) is activated in the first cycle, capacities of 600 and 560 mAh g^−1^ are maintained after 150 cycles at rates of 0.5 C and 1 C, respectively. In contrast, the Li–S cell without the HC−In exhibits slightly lower capacities of 247 and 205 mAh g^−1^ after 150 cycles. The specific capacity is approximately twice as high when the HC−In is used compared with that when it is not used. With the FCCS, the Li_2_S material dissolves in the electrolyte between the Li and S electrode materials, resulting in a lower capacity than that of the HC−In material after 150 cycles. In addition, the Li_2_S material with the FCCS exhibits a low specific capacity. Therefore, the results show that the HC−In is effective in maintaining capacity stability when used as an interlayer.

**Figure 6:**
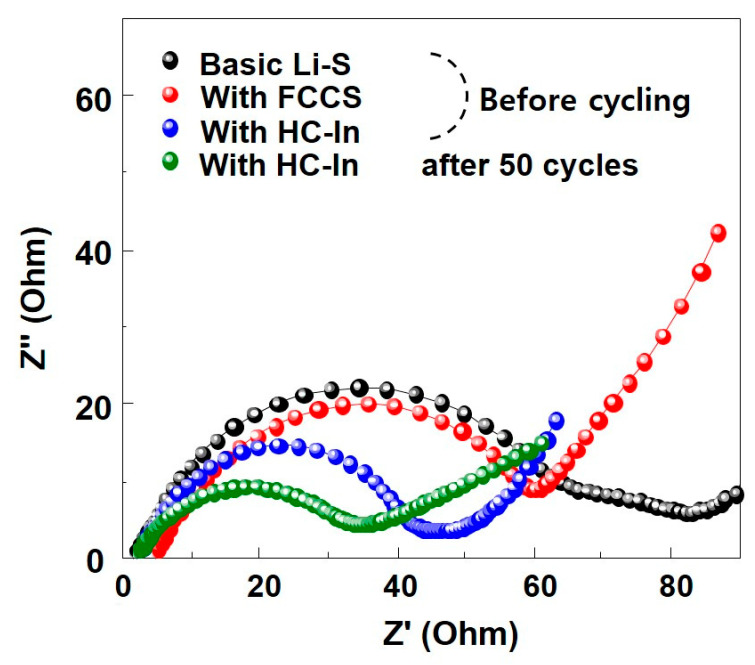
EIS results of the cells with the FCCS and HC−In before cycling and after the 50th cycle.

Electrochemical impedance spectroscopy (EIS) was performed to understand the impact of an interlayer on the electrochemical kinetics of the battery. Conductive carbon paper is known for reducing interfacial resistance by intercalating the intermediate layer, which effectively facilitates the rapid electrochemical reaction of Li–S cells by providing abundant reaction sites supported by carbon [29]. This observation is consistent with the EIS results shown in Figure 6. The measurement frequency range was set from 10^−1^ Hz to 10^−7^ Hz. The impedance plot for the cells, both with and without an interlayer, initially displayed a semicircle in the high−to−intermediate-frequency range, followed by an inclined line in the low−frequency range. The high-frequency region represents the resistance (R_s_) for ion movement within the electrolyte. In the mid-to-low-frequency range, the semicircle signifies the charge-transfer resistance (R_ct_) due to ions moving at the interface through electrochemical oxidation/reduction reactions. Before cycling, the charge-transfer resistance was significantly lower in both the FCCS and HC−In samples compared with the basic Li–S battery (approximately 40–50 Ω). This reduction in R_ct_ is attributed to the highly conductive interlayers, which enable low electrochemical reaction resistance. The EIS results demonstrated that the charge-transfer resistance of the battery decreased when using the HC−In or FCCS, indicating more efficient electron movement. The resistance values indicate that the HC−In outperformed the FCCS, with the resistance decreasing even after cycling the HC−In cell 50 times. This phenomenon occurred after electrochemical reactions and exhibited distinct characteristics. Note that the HC−In sample exhibited only one semicircle even after cycling, and its low R_ct_ value indicates fast-reaction kinetics. Based on these results, the HC−In interlayer serves as a current collector for the low-conductivity sulfur cathode, enhancing the utilization of the active material and, thereby, contributing to an increase in the specific capacity of the cell.

The results obtained in this study using the HC−In are consistent with previous findings using multiwalled carbon nanotubes [15,16], microporous carbon paper [17], and carbon paper interlayers [18,19]. However, the preparation process for pyrolyzed hanji is straightforward and convenient compared to the methods used for manufacturing intermediate films of multiwalled carbon nanotubes or other carbon papers.

**Figure 7:**
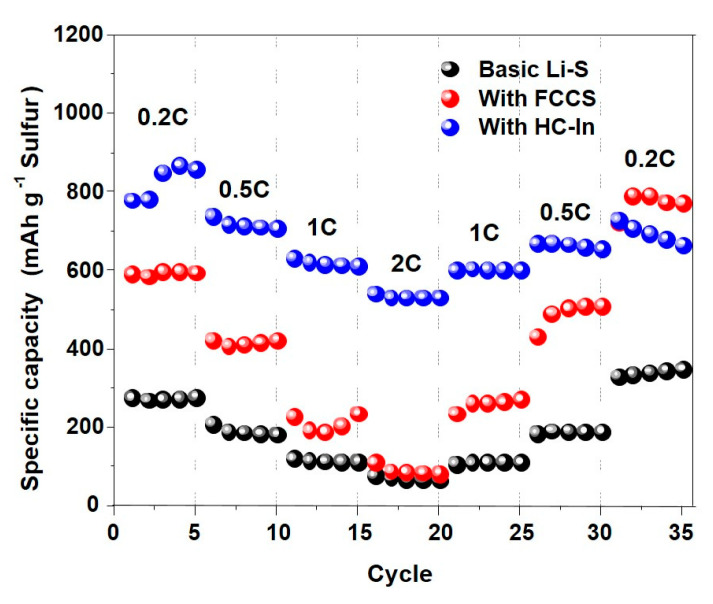
Rate capability of the Li–S cells with and without the FCCS and HC−In.

Figure 7 shows the C-rate characteristics with and without the use of the HC−In. The discharge capacities of the Li–S cells using the HC−In are 920, 690, 600, and 510 mAh g^−1^ at 0.2 C, 0.5 C, 1 C, and 2 C, respectively. An approximately 350% improvement in capacity is observed compared to cells that do not utilize the interlayer. These results indicate that the incorporation of the HC−In increases the rate capability of the Li–S cells. With the FCCS, the discharge capacity changes rapidly as the rate varies. Based on the 2 C standard, the discharge capacity is similar to that of the standard cell. Thus, the HC−In is more advantageous than the FCCS when a large-capacity battery is required under instantaneous high-current conditions.

**Figure 8:**
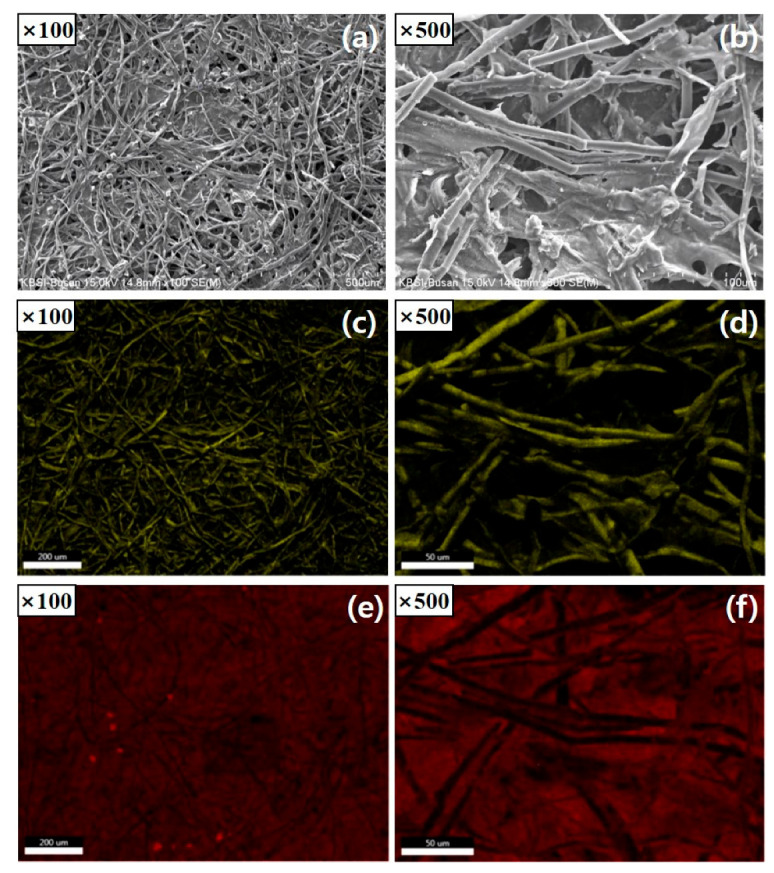
(**a**,**b**) SEM images and EDX maps of (**c**,**d**) C and (**e**,**f**) S in the HC−In C intermediate layer.

Figure 8 shows an SEM image of the fiber shape and porous structure of the HC−In carbon intermediate layer. Figure 8a,b show images captured at magnifications of 100× and 500×, respectively. Figure 8c, 8d show the images for carbon, and Figure 8e,f show the images for sulfur.

The porosity of the intermediate carbon layer enables electrolytes to permeate during cycling [6,11,15]. Additionally, the pores in the C paper can minimize the presence of polysulfide intermediates [11]. Figure 8b shows the accumulation of active substances. This indicates that the carbon-fiber band in the HC−In serves as a matrix for adsorbing and retaining polysulfide species, in addition to acting as a secondary redox reaction site. The elemental mapping of carbon and sulfur in the HC−In intermediate layer (Figure 8c–f) shows that S is uniformly distributed throughout the intermediate layer and undergoes a reaction for 50 cycles. Notably, in the absence of the HC−In, the irreversible capacity loss limits the charge-transfer reaction and ion transport within the battery. Carbon adsorbs the polysulfides formed during charging and discharging, ensuring excellent re-usability and reversibility of the sulfur cathode material. The good distribution of the carbon material near the fiber indicates that the fiber is not heavily coated with sulfur/sulfide, resulting in good conductivity in the cathode region, even during extended cycles.

We compared the data from several previously reported papers. (Table 1). The HC−In data demonstrated slightly better or similar performance compared to other reported materials. Although the initial capacity of HC−In cells may not be as clearly good as other cells, we observed that their performance remains stable when comparing cycles. Subsequently, we will take advantage of the HC−In interlayer insertion to increase cycle life and improve the experiment to investigate its effect on high loading mass.

## 4. Conclusions

Hanji was manufactured by a simple pyrolysis process to create porous carbon paper. When used as an interlayer between the sulfur cathode and separator, the synthetic carbon paper significantly increased the specific capacity, cycle stability, and speed of the Li–S battery. The improved ion mobility and electron conductivity of hanji make it an effective intermediate support for the utilization of active materials. The cathode surface effectively made contact with the flexible carbon paper, facilitating the movement of polysulfide intermediates and providing an insulated electron pathway through S/Li_2_S. After conducting experiments using the HC−In interlayer, the initial capacities were 935.26 mAh g^−1^ at 0.5 C and 873.11 mAh g^−1^ at 1 C. After 150 cycles, the capacities decreased to 600 mAh g^−1^ and 560 mAh g^−1^, respectively. This result demonstrates higher retention in the Li–S cell with the HC−In compared to the initial capacity when compared with other cells. Thus, hanji, as a biomass material, significantly improved the cycling performance of Li–S batteries. Consequently, the use of carbon paper and optimized cell topologies can improve the performance of sulfur electrodes and address various issues associated with Li–S batteries.

## Figures and Tables

**Table 1:** Applied interlayers and their properties for Li–S battery.

Description	Sulfur Content (%)	Mass Loading	Initial Capacities (mAh g^−1^)	Capacity (mAh g^−1^)	Ref.
CVD graphene	63	8 μg cm^-2^	1039	C dis = 619 mAh g^−1^ Cycle number = 100 Rate = 0.2 C	[30]
Nonwoven carbon fiber fabric	70	-	1486	C dis = 858 mAh g^−1^ Cycle number = 100 Rate = 0.1 C	[31]
Silkworm excrement-derived porous carbon	80	-	1295.1	C dis = 786 mAh g^−1^ Cycle number = 100 Rate = 0.2 C	[32]
Vanadium nitride nanodots CNT	70	~0.6 mg cm^-2^	1097	C dis = 432 mAh g^−1^ Cycle number = 400 Rate = 0.1 C	[33]
Yeast	70	~0.46 mg cm^-2^	800.2	C dis = 642 mAh g^−1^ Cycle number = 100 Rate = 0.1 C	[34]
HC−In	70	~2.2 mg cm^-2^	935	C dis = 604 mAh g^−1^ Cycle number = 100 Rate = 0.5 C	This work

## Data Availability

The data presented in this study are available on request from the corresponding author.
